# Pharmacological and Physiological Correlates of the Bidirectional Fear Phenotype of the Carioca Rats and Other Bidirectionally Selected Lines

**DOI:** 10.2174/1570159X20666221012121534

**Published:** 2023-07-10

**Authors:** Yury V. Lages, Laura Balthazar, Thomas. E. Krahe, J. Landeira-Fernandez

**Affiliations:** 1 Department of Psychology, Pontifical Catholic University of Rio de Janeiro, Rio de Janeiro, Brazil;; 2 Department of Physiological Sciences, Laboratory of Neurophysiology, Biology Institute, State University of Rio de Janeiro, Rio de Janeiro, Brazil

**Keywords:** Breeding lines, fear, anxiety, stress, serotonin, dopamine, metabolism

## Abstract

The Carioca rat lines originated from the selective bidirectional breeding of mates displaying extreme defense responses to contextual conditioned fear. After three generations, two distinct populations could be distinguished: the Carioca High- and Low-conditioned Freezing rats, CHF, and CLF, respectively. Later studies identified strong anxiety-like behaviors in the CHF line, while indications of impulsivity and hyperactivity were prominent in the CLF animals. The present review details the physiological and pharmacological-related findings obtained from these lines. The results discussed here point towards a dysfunctional fear circuitry in CHF rats, including alterations in key brain structures and the serotoninergic system. Moreover, data from these animals highlight important alterations in the stress-processing machinery and its associated systems, such as energy metabolism and antioxidative defense. Finally, evidence of an alteration in the dopaminergic pathway in CLF rats is also debated. Thus, accumulating data gathered over the years, place the Carioca lines as significant animal models for the study of psychiatric disorders, especially fear-related ones like anxiety.

## 1 INTRODUCTION

Animal models constitute an important tool to reproduce characteristics of human psychiatric disorders in laboratory animals. For this purpose, they must be able to reflect physiological and behavioral features associated with specific emotions, the etiology of disorders, and the responses to pharmacological treatments [1]. Also, due to the low cost, speed, and experimental reproducibility, animal models have significantly contributed to the discovery of novel psychotropic drugs and the understanding of the neurobiology of psychiatric diseases [2].

The modeling of anxiety disorders in animals is of great importance given the health burden caused by them. Anxiety disorders are the second most prevalent mental health condition in the world, affecting 3.6% of the population, being surpassed only by depression, which affects 4.4% of people [3]. Animal models of anxiety date to the early 1930s when Calvin Hall described defecation and urination in rodents in the open field (OF) arena as a relevant behavioral parameter to measure anxiety-like states [4]. Later, many other animal models of anxiety were developed based on the bidirectional breeding of animals given their performance on ethological tests [5-7]. The tasks are constructed around the innate and conflicting urges of rodents to explore new spaces and avoid open, exposed and brightly lit areas where they could be more vulnerable to environmental threats [8]. In general, in such conflicting approach-avoidance tasks, rodents with an anxious phenotype tend to spend more time in closed and “safe” areas of the testing apparatus than their respective controls [9]. It is worth noting that models based on different paradigms reflect distinct forms of anxiety [10], which might reflect their underlying altered neural circuitry. Among the latter, we can cite the recruitment of the septohippocampal system to produce the cognitive component of fear [11, 12], and the amygdala-ventral periaqueductal gray (PAG) circuit which is involved in the regulation of inhibitory behavior in response to innate or conditioned aversive stimuli [13, 14]. Moreover, depending on whether fear exposure is acute or chronic, different neural circuits are employed. In acute fear, the basolateral amygdala (BLA), central amygdala (CeA), and brainstem circuit are mainly responsible for the behavioral outputs, while during sustained fear, projections from the BLA directly to the bed nucleus of the stria terminalis (BNST) mediate physiological responses, such as increased heart rate and blood pressure, activation of the stress circuitry and muscle contraction. This sidetrack is also used during the increase in corticotropin-releasing hormone (CRH), one of the main components of the stress pathway [15-17].

An important caveat concerning ethology-based animal models is that it may be difficult to differentiate the anxiety phenotype from novelty-seeking, exploring, or impulsive behaviors [9]. To circumvent this limitation, our group and others have developed animal models of anxiety that rely on associative fear learning mechanisms [18]. In particular, the Carioca rat lines were developed based on pavlovian fear conditioning, a method that consists of the association of two stimuli: an unconditioned stimulus (US) and a discrete stimulus which, as it is associated with the US, becomes the conditioned stimulus (CS). In animal models of anxiety, the US is most commonly a footshock and the CS can be a tone or visual cue, or the surrounding context itself [19]. According to Landeira-Fernandez [20], context-conditioned fear represents one of the simplest and fastest ways to produce aversive learning. As the initially neutral environment is connected to the footshock, it evokes fear and defensive responses. When faced again with the CS, freezing is the most likely response in anticipation of a potential US and it has been largely considered an anxiety-like behavior [19].

The Carioca lines were derived from bidirectional selective breeding based on their freezing behavior in response to contextual cues from the experimental chamber in which animals, 24 hours before, had been exposed to mildly aversive electrical footshocks. As early as the third generation, male animals started to show reliable differences in freezing responses (*i.e*., high and low freezing groups), indicating a strong heritable component related to fear learning [21]. Thus, two lines with opposite freezing patterns were established: the CHF (Carioca High-conditioned Freezing), with strong defensive reaction traits, and the CLF (Carioca Low-conditioned Freezing), a lineage that displays low freezing responses to contextual fear. Later studies [22] showed that clear differences in freezing behavior between CHF and CLF were reliable and still present after the 12^th^ generation and that the marked high freezing of the CHF line (males > females) is maintained 60 days after contextual fear conditioning, suggesting a long-term memory consolidation of contextual fear conditioning in this line. More recently, the distinctive freezing phenotypes of CHF and CLF rats were confirmed in the 36^th^ generation of the two lines [23]. The development and strengthening of opposing freezing responses in the Carioca lines over successive generations are demonstrated in Fig. (**1**) [21-26].

Based on the behavioral characterization of the Carioca lines, it has been proposed that the CHF rats represent a model of generalized anxiety disorder (GAD), with CLF as their experimental counterpart [22, 24]. In this review, we discuss the pharmacological and physiological findings made over the years using the Carioca lines, emphasizing their association to behavioral outcomes as well as their relevance in comparison to other distinguishable models of psychiatric disorders. Furthermore, we aim at substantiating the establishment of the CHF as an animal model of GAD and exploring evidence of altered locomotory mechanisms in CLF rats.

## 2 BENZODIAZEPINE TREATMENT

Willner [27] defined that an animal model of a psychiatric disorder must be responsive to pharmacological treatments used in the human clinic. For anxiety disorders, benzodiazepines are the pharmacological gold standard for the validation of animal models given their efficacy in the human clinic and their biological mechanism [28, 29]. Benzodiazepines bind to the central γ-aminobutyric acid type A (GABAA). Activation of GABAA receptors leads to hyperpolarization and inhibition of neuro-transmission, which have been associated with the anxiolytic effects of benzodiazepines [30]. The evaluation of the pharmacological effects of these drugs is often made using the elevated plus maze (EPM), a behavior paradigm widely used to measure innate fear and anxiety-like responses in rodents [31]. Cavaliere *et al.* (2020) [25] administered 0.25, 0.5, and 1.0 mg/kg of midazolam to CHF, CLF, and control male animals 30 min before testing their behavior in the elevated plus-maze (EPM) for 5 min. They found that CHF rats constitutively had fewer entries and spend less time in the open arms of the EPM compared to both control and CLF rats. Administration of 0.5 mg/kg midazolam had an anxiolytic effect in CHF and control groups by increasing the number of entries and time spent in the open arms of the EPM. This effect was not linked to a reduction in the overall locomotor activity in either group, which says against a locomotory effect of the drug. It is worth noting that the CLF group displayed an opposite behavioral pattern compared to CHF rats - a high number of entries and a great amount of time spent in the open arms of the EPM. Follow-up studies confirmed these behavior differences between the groups, *i.e*. the number of entries and time spent in the open arms of the apparatus is lower in CHF compared to CLF male and female animals [32-35] (Table **1**).

Similar to the EPM, the open field (OF) is a behavioral test commonly employed to measure innate fear and overall mobility in rodents [36]. Lages *et al.* (2021) [37] showed that the CHF male rats have a smaller number of entries in the center of the apparatus in comparison to the CLF group, confirming that CHF rats display an increased anxiety behavioral phenotype. The authors also observed that, compared to CHF rats, CLF male animals expressed a significantly high locomotor activity, which is in accordance with a previous study [38]. Whether the administration of benzodiazepines has an anxiolytic effect on the OF behavior of CHF rats remains to be investigated.

Basal differences between innate and conditioned fear and the effects of benzodiazepines have been demonstrated in other animal models of anxiety. The Roman high- (RHA) and low-avoidance (RLA) outbred rat lines were bidirectionally bred for the respectively rapid or poor acquisition of active avoidant behavior. Consequently, the RLA rats are more fearful/anxious than their RHA counterparts [39]. When treated with 3 mg/kg diazepam, RLA rats displayed a decreased freezing behavior in the shuttle box as well as reduced hippocampal content of benzodiazepine-like molecules [40]. Moreover, compared to the RHA line, RLA rats showed lower specific diazepam binding in the cortex, striatum, hippocampus, thalamic region, and pons-medulla [41]. RLA rats also showed a significant reduction in the allosteric interactions between GABA and [3H]zolpidem (a Type I selective agonist) binding sites in the cortex [42].

Another well-known and studied animal model of anxiety is the HAB/LAB lines, developed based on the bidirectional breeding of Wistar rats that differ markedly in their anxiety-related behavior in the EPM. In contrast to the LAB line, HAB rats show more pronounced anxiety-like and stress-related responses [43]. One mg/kg of diazepam increased the constitutively low percentage of entries into and time spent on the open arms of the EPM in HAB rats while mildly affecting the behavior of the LAB rats [44, 45]. Moreover, diazepam administration has also been shown to promote the increase of immobility of HAB rats during the resident intruder test [45] and their paw withdrawal latency in a nociception test [46]. Additional studies with the HAB/LAB rats didn’t find evidence suggesting that the pharmacology of GABAA receptors accounts for the different sensitivity to the anxiolytic effects of benzodiazepines; however, one could not exclude possible variations in the function of GABAA receptors [47]. Thus, compared to the evidence found in other lines, it is yet to be studied whether the Carioca lines exhibit differences in benzodiazepine binding sites and their possible association with specific fear behavior responses.

## 3 SEROTONINERGIC ANTAGONISM

Despite its proven efficacy in treating anxiety [48], the use of benzodiazepines presents important risks such as addiction, abuse [49-51], dementia, and cognitive impairment [52], which make them the second or third in line for the treatment of anxiety [53]. Given the close association in the neural background between anxiety disorders and depression [54], antidepressants are currently the first choice of drugs to treat anxiety [55]. Since the first evidence in the 1950s that altered monoaminergic pathways were implicated in the dysfunctional behavior of mood disorders, serotoninergic mediation has been the primal target of antidepressants [56].

From a physiological point of view, serotoninergic neurons of the dorsal raphe nucleus (DRN) project to key structures involved in fear, such as the amygdala, hippocampus, and prefrontal cortex (PFC), modulating defensive behaviors through learning mechanisms [57]. The regulatory role of serotoninergic efferents from the DRN is involved in the development of fear responses and an anxiety profile [58-61]. Thus, the pharmacological inhibition of DRN neurons, or serotoninergic receptors in the amygdala, prevents the development of stress-induced anxiety [62]. The serotonin receptors are also an important site of action of antidepressants. Studies showed that treating humans or mice that had a depressive disorder with selective serotonin reuptake inhibitors (SSRIs) led to lower expression of 5-HT2A and 5-HT2C [63, 64]. Furthermore, the increase in 5-HT caused by the inhibition of serotonin transporters activated these receptors located in the locus coeruleus (LC) and the ventral tegmental area (VTA), leading to a reduction in the firing of noradrenergic and dopaminergic neurons and exacerbation of depressive behaviors [65, 66].

Leon *et al.* (2017) [32] administered ketanserin, a 5-HT2A receptor antagonist, to male Carioca rats to evaluate their behavior in the EPM and the contextually conditioned fear test. Injections were made either systemically (i.p, 0.5 mg/kg) or intra the infralimbic (IL) and the prelimbic cortices (PL) (5 nmol/ml, 0.5 ml/side) 30 min before behavioral testing. The IL and PL regions of the ventral medial PFC cortex exert different functions on fear regulation. The descending projections of the IL to the amygdala play an important role in the extinction of conditioned fear [67-69]. Studies showed that the inactivation of the IL impaired the consolidation and retrieval of the extinction of fear conditioning [70, 71]. Neurons in the PL play an excitatory role in conditioned fear behavior. On the other hand, the efferents of PL neurons on the BLA exert an excitatory role in conditioned fear behavior [67]. Reports indicated that pharmacological inhibition of the PL reduces the expression of conditioned fear [72]. Results with the Carioca lines show that the systemic and intra IL administration of ketanserin produced anxiolytic effects in CHF and anxiogenic effects on CLF rats as measured in the EPM; also, the same opposing results were observed in the contextual conditioned fear test after IL injections. The intra PL infusion of the 5-HT2A antagonist produced only anxiolytic effects on CHF rats. No locomotory alterations were observed. The authors associate the different responses of CHF and CLF male rats to differences in 5-HT2 receptor expression in the limbic cortices [32].

Altered serotoninergic pathways have also been identified in other models of anxiety disorders. Umriukhin *et al.* (2002) [73] showed that the serotonin content in the paraventricular nucleus of the hypothalamus (PVN) and the dorsal hippocampus was equivalent in HAB and LAB rats under basal conditions, but differed after behavioral tests. The forced swim stress induced an increase in serotonin release in the PVN of both HAB and LAB rats, and also in the dorsal hippocampus of the LAB group. A similar change in hippocampal serotonin of the LAB rats was also observed after the elevated platform challenge. Nonetheless, basal hippocampal 5-HT1A receptors expression and overall expression of serotonin transporter binding sites are increased in HAB compared to LAB rats [74]. In the Roman low-and high-avoidance (RLA and RHA, respectively) lines the activity of tryptophan hydroxylase, the main enzyme to the synthesis of the serotonin activity, in the midbrain (but not in the cortex, hippocampus, or hypothalamus) was lower in RLA rats than in RHA rats. Also, hypothalamic binding sites of citalopram and the cortical expression of 5-HT2A receptor were lower in RLA rats [75]. Further studies show contrasting results. Visser *et al.* (2015) [76] found no differences in 5-HT2A receptor binding sites in the PFC and hippocampus of both lines. Fomsgaard *et al.* (2018) [77] observed greater 5-HT2A binding in the PFC of RHA rats, while no difference in binding was observed in the striatum. As the 5-HT2A gene expression was unchanged in PFC, the authors attributed the binding differences to post-translational regulation.

## 4 DOPAMINERGIC CIRCUITRY

Although serotonergic mechanisms might be involved in the alterations that are observed in CHF male rats [32], no such evidence yet supports such a hypothesis for the CLF line. Based on the involvement of other monoamines in fear regulation and mood disorder [65, 66], and the indications of impulsivity and motor alterations in CLF male animals [37], the dopaminergic mediation of CLF’s behavior was further investigated. Dopamine takes part in fear acquisition and extinction [78, 79], and is a central component in attention-deficit/hyperactivity disorders (ADHD) [80-83].

A recent study from Lages *et al.* (2021) [84] investigated the motor behavior and responses to conditioned fear of CLF male rats following treatment with haloperidol and methylphenidate. Haloperidol is a dopamine D_2_ receptor antagonist and a typical antipsychotic medication [85]. Methylphenidate is a psychostimulant that is commonly used for the clinical treatment of ADHD. It acts as a norepinephrine-dopamine reuptake inhibitor (NDRI), with a higher affinity for the dopamine transporter (DAT), resulting in increases in alertness and attention [86]. In the Lages *et al.* (2021) study [84], haloperidol (i.p., 0.5, 1.0, and 1.5 mg/kg) was administered to induce catatonia characterized by rearing and immobilization, whereas methylphenidate (i.p., 0.5, 1.0 and 2.0 mg/kg) was injected 25 and 30 min before the OF and the testing phase of context fear conditioning, respectively. The results showed that compared to randomly bred male rats (control), CLF rats exhibited lower sensitivity to haloperidol-induced catatonia and an increase in freezing and motor behavior in response to methylphenidate. Given the molecular target of these drugs, the distinguished behavior of CLF rats, and the implication of dopaminergic mechanisms in ADHD, the authors suggest that this line may have inherent background alterations in the dopaminergic circuitry.

Altered dopaminergic circuitry is present in several animal models of ADHD. For example, the knockout mice for the p35 gene display spontaneous hyperactivity, a distinguishing characteristic of ADHD [87]. In these animals, there is a decrease in dopamine uptake and turnover due to the lower availability of DAT on the cellular membrane of neurons in the striatum [88]. Moreover, it was also observed that these mice have a loss of descending mPFC outputs to the nucleus accumbens and an increase of dopaminergic afferents projecting from the ventral tegmental area to the mPFC. Following treatment with methylphenidate, the mice display altered motor behavior which was linked to changes in PFC neurotransmitter content and the restoration of dopamine content in the striatum [87, 89]. Another animal model of ADHD is the coloboma mouse line; their spontaneous hyperactivity has been linked to the activity of D2-like receptors. While depletion of D2 receptors and amphetamine can reduce the hyperactive behavior of coloboma mice, administration of a D2 dopamine receptor-selective antagonist blocked such amphetamine-induced reduction in locomotor activity [90]. Lastly, in the Naples High Excitability rats, selectively bred for their performance in the Làt maze, intranasal application of dopamine had a dose-dependent effect of reducing hyperactivity and improving attention in both Làt and radial mazes [91]. Additionally, Leffa *et al.* [92] conducted a meta-analysis of the behavioral effects of methylphenidate in Spontaneously Hypertensive Rats (SHR), the most widely studied animal model of ADHD. Their results show that this drug had a significant and reliable effect on increasing the attention of SHR animals in a variety of behavioral tests, namely the Làt-maze, attentional set-shifting test (ASST), Y-maze, five-trial inhibitory avoidance test, orienting behavior, among others. This effect can be correlated to an imbalance of dopaminergic mediation - a previous report [93] shows that both tyrosine hydroxylase (TH) and DA transporter gene expression are significantly and transiently reduced in the SHR midbrain during the first month of postnatal development. TH catalyzes the conversion of the amino acid L-tyrosine to L-3,4-dihydroxyphenylalanine (L-DOPA), the precursor of dopamine. Moreover, DA uptake activity is significantly reduced in synaptosomes of the SHR striatum during the same period [93].

## 5 NEUROPHYSIOLOGICAL STUDIES

Central to the processing of fear are the amygdala and the hippocampus [94]. As mentioned before, these structures are modulated by descending projections from the PFC and ascending projections from the DRN. In conditioned fear, the hippocampus relays contextual and mnemonic information, while the amygdala evaluates the valency of the stimulus [95]. As the hippocampus signals to the amygdala *via* either the PL or the IL cortices, the BLA gathers information from the thalamus and sensory cortical regions. The processed information is then relayed *via* CeA to the BNST and the PAG that produce behavioral responses [94].

Galvão *et al.* (2011) [96] tested whether alterations in the anticipatory anxiety of the Carioca lines might be related to the expression of panic responses mediated by the PAG. Based on previous evidence that anxiety has an inhibitory effect on panic attack-like behavior [97], the authors applied electrical stimulation to the dorsal PAG (dPAG) and measured the freezing and escaping responses of animals previously conditioned to aversive contextual cues. Contrary to expectations, the results showed that the aversive threshold for producing freezing and escape reactions following dPAG electrical stimulation was higher in CHF male animals than in CLF ones. Moreover, at the escape threshold, the freezing of CHF rats was increased immediately after dPAG electrical stimulation compared to CLF animals. Considering that dPAG activation involves different neurocircuits than those of anxiety and is associated with panic responses [98, 99], these findings demonstrate that anticipatory anxiety is higher in CHF rats and suggest that may contribute to the development of panic disorder triggered by panic attacks. Even though the midbrain and anterior cingulate cortex are mostly activated during panic behaviors [100], other components of the fear circuitry are also involved, such as the hippocampus, basal ganglia, insula, and, most importantly, the amygdala [100, 101]. Descending inhibitory projections from the amygdaloid complex reach the dPAG, which in turn might inhibit defensive unconditioned reactions triggered by this structure. Conversely, ascending projections from the dPAG to the amygdaloid complex modulate fear conditioning and might represent one of the main pathways affected in panic disorder [102, 103].

The first published study of the Carioca lines evaluated the role of the amygdala on their fear conditioning behavior [21]. In this study, three weeks after training and testing sessions of contextual fear conditioning, CHF and CLF male animals received bilateral electrolytic lesions in the amygdala. Then, a week after surgery, the animals were re-tested for the expression of conditioned fear responses. The results showed that freezing was reduced in both lines in comparison to their respective counterparts that underwent sham surgery, demonstrating the important role of the amygdala in the consolidation of fear memories and the expression of defensive responses in these animals. In the Roman rat lines, the opposite behavior of RHA and RLA rats in the one-way avoidance test is correlated with cellular density in the BLA; RLA rats show increased neural density in several areas of the BLA compared to RHA animals [104]. Moreover, RLA rats showed greater hippocampus and amygdala volumes than untreated RHA rats which in turn is related to higher anxiety and post-stress hormone responses [105, 106]. Furthermore, another study revealed that bilateral electrolytic lesions of the CeA increased the number of trials needed to reach five consecutive avoidance responses in one-way avoidance tests, thus abolishing the between-strain performance differences, *i.e*. RHA animals displayed a lower time to five consecutive avoidance responses compared to RLA ones [107].

Differences in the amygdala and other regions of the fear circuitry between the Carioca lines were also evaluated in terms of NMDA receptor subunits and glutamate transporters. The glutamate pathway exerts an important role in neural plasticity, cell proliferation, and migration, including memory and learning processes, such as the acquisition and extinction of fear-conditioning [108, 109]. Notwithstanding, it is linked to anxiety and depressive disorders. Increased levels of serum glutamate and serine, in addition to an altered function of glutamate decarboxylase, have been identified in human subjects with mood disorders [110-113]. Moreover, mice knockout for the glutamate receptor Delta-1 manifested anxiety and depressive-like behavior [114]. Regarding the Carioca lines, Réus *et al.* (2015) [115] identified the following differences in CHF male rats in comparison to control and CLF animals: i) in the amygdala, there are increased levels of NMDAR1, NMDAR2 (in the Chronic Mild Stress - CMS - group), and excitatory amino acid transporter 1 (EAAT1) and decreased levels of NMDAR2, NMDAR2B, and excitatory amino acid transporter 2 (EAAT2); ii) in the PFC there were higher levels of NMDAR1A, NMDAR2B, and EAAT-2; iii) in the nucleus accumbens, an increased expression of NMDAR2B; and iv) in the hippocampus, there was higher expression of NMDAR1, NMDAR2B, and EAAT2. In CLF rats, only lower levels of NMDAR2 were identified in the nucleus accumbens and higher levels of NMDAR2B in the hippocampus. In a previous study by Goulart *et al.* (2011) [38], the expression of different glutamatergic receptor subunits was evaluated in the hippocampus of Carioca male rats. The results obtained from western blot analysis showed that the expression of the AMPAR GluA1 subunit was increased in CHF animals, whereas GluA2 subunits were higher in CLF ones. Little is known about the role of AMPAR subunits in anxiety-like behavior and stress response, however, AMPAR agonists and antagonists can produce anxiogenic and anxiolytic effects, respectively [116, 117]. Therefore, Goulart and colleagues [38] associated the imbalance of GluA2/GluA1 in CLF male animals with an increased AMPAR instability and consecutively to greater plasticity. Moreover, the study showed that the expression of the NMDA receptor GluN1 subunit in the hippocampus was higher in both lines in comparison to control, randomly bred, animals. NMDAR GluN2A subunit was decreased in CHF rats, while GluN2B subunits were lower in the CLF group. NMDAR GluN2A and GluN2B subunits play different roles in synapses. The GluN2A subunit is activated by highly correlated stimuli through the rapid activation and inactivation of NMDA channels, while the GluN2B subunit allows the maintenance of excitatory currents [118]. Ontogenetically, the GluN1/GluN2B expression is higher in early development and later there is a shift to GluN1/ GluN2A following the maturation of the nervous system. Therefore, the GluN2A/Glun2B ratio is considered an index of synaptic maturation [118]. CHF animals displayed a more immature profile of NMDARs, given the predominance of GluN1/GuN2B subunits. As a consequence, the activation of hippocampal neurons would be potentiated by external stimuli, such as stress [119]. Indeed, recent results from our group showed that the CHF line is more susceptible to chronic stress [37]. While the chronic unpredictable mild stress (CUMS) protocol produced subtle behavior alterations in the CLF line, it induced a shift and exacerbation of defensive coping strategies in CHF male rats. Specifically, freezing of CHF rats was increased in innate fear behavioral tests, such as the OF and the elevated T maze, while active responses were triggered in the contextual fear conditioning and forced swim tests (FST). In addition, palatable food, rich in sugar and fat, was demonstrated to attenuate the alterations caused by chronic stress, particularly in the CHF line [26]. Future studies are needed to investigate whether these alterations are involved with differences in the expression of glutamate receptors and transporters in CHF and CLF rats after chronic stress targeting brain regions involved with fear processing, such as the PFC, and amygdala, hippocampus, and nucleus accumbens [115].

Although no differences were found in the cellular density of hippocampal CA1 and CA3 areas or the dentate gyrus (DG) between the two Carioca lines [33], the number of neuroblasts is significantly reduced in the DG of CHF male rats, which also display deficient dendritic morphology [120]. Moreover, CHF animals showed reduced release of GABA and an increased number of dendritic spikes and expression of brain-derived neurotrophic factor (BDNF) precursor [120]. The use of SSRIs is well known for stimulating the release of neurotrophins such as BDNF, glial cell line-derived neurotrophic factor (GDNF), and vascular endothelial growth factor (VEGF), which in turn, promote the proliferation, maturation, and survival of hippocampal DG cells [121, 122]. Anxiety and depressive disorders have been linked to altered neurogenesis and neuronal plasticity, which are associated with serotoninergic transmission and can be partially reversed by treatment with SSRIs [123-125]. 5-HT2 and 5-HT2C antagonists lead to an increase in cellular proliferation [126-128], potentially by the activation of the ERK1/2 pathway [129].

Regarding the HAB/LAB lines, the survival of newly generated hippocampal cells was found to be significantly lower in 43-day-old HAB than in LAB rats of the same age, which may be linked to the failure to increase placental 11-beta-HSD2 activity after stress. This enzyme catalyzes the inactivation of maternal corticosterone, serving as a physiological 'barrier' to maternal glucocorticoids. Thus, HAB rats displayed increased susceptibility to stress [130]. The expression and activity of proteins involved with mood disorders are also different in the hippocampus of these lines. The altered metabolism of sphingolipids such as sphingomyelin and ceramide has been linked to the pathogenesis of major depressive disorder [131]. Likewise, results showed that acid and neutral sphingomyelinases (ASM, NSM) and ceramidases (AC, NC) have increased activity not only in the ventral hippocampus of HAB rats but also in the lateral septum, hypothalamus, ventral and dorsal mesencephalon [131]. Importantly, anxiety-like behavior in the EPM test was found to be correlated with the ASM activity in the ventral hippocampus, the amygdala, and dorsal mesencephalon of female HAB rats [132]. NSM activity levels in the dorsal mesencephalon were also correlated with depressive behavior in these animals [132]. Moreover, neuropeptides relevant for anxiety- and depression-related behaviors were found to be altered in the hippocampus of HAB/LAB lines. Particularly, elevated levels of calcitonin gene-related peptide (CGRP) were detected in the hippocampus of HAB rats, which in turn, correlated to their anxiety-like behavior in the EPM and depressive-like responses in the FST [133]. CGRP is known to be related to anxiety disorders since intracerebroventricular administration and BNST infusions of CGRP were found to produce anxiogenic behaviors in rats [134-136].

Other brain regions of the Carioca lines have also been compared for differences in neuronal activity. A recent study by León *et al.* (2020) [137] showed that compared to control animals, CHF male rats have increased Fos expression in the locus coeruleus (LC), PVN, and lateral portion of the septal area and decreased Fos expression in the medial portion of the septal area, DG, and PL. The LC and the PVN are particularly relevant for the activation of the HPA axis [138-141] and there’s been consistent evidence that generalized anxiety activates both the HPA axis and sympathoadrenal axis [142, 143], of which the latter is also regulated by norepinephrine efferent activity from the LC [144]. The hyperactivation and hypoactivation of the lateral and medial portions of the septal area, respectively, in CHF are consistent with a previous report that showed that LS lesions decreased and MS lesions increased conditioned freezing in response to contextual cues that were associated with footshocks [145]. Also, communication between the medial septal area and the DG produces glucocorticoid-induced feedback inhibition of the HPA axis [141, 146, 147]. Thus, decreased activity in the medial septal area and the DG can elicit increased neuronal activity in the PVN and an exacerbated HPA neuroendocrine response to threatening stimuli and stress. In contrast to the important role of the prelimbic cortex in the acquisition and expression of conditioned fear [148, 149], including in CHF male rats as mentioned before [32], lower Fos activity was observed in the PL of these animals. The authors hypothesized that the differences in neuronal activity in this region and the high-freezing responses of CHF rats may come as a result of an inhibitory feedback signal to the HPA axis [150] *via* activation of the DG [69]. However, a closer investigation is needed to verify the actual contribution of the PL to the behavioral pattern of these animals.

León *et al.* (2020) [137] also identified a distinct pattern of Fos activation in CLF male animals. In comparison to control rats, the CLF line exhibited a decrease in Fos expression in the PVN, PL, and BLA and an increase in the cingulate and perirhinal cortices. Hypoactivity in the PVN and BLA is consistent with the low-conditioned freezing characteristic of CLF rats. The authors also associated such behavioral traits with the weak activation of the PL. As an important mediator of conditioned fear behavior, including through its connection to the amygdala, the hypoactivation of the PL has been linked to decreased defensive responses [72, 151]. The perirhinal and the cingulate cortices are also involved in fear regulation. When the external stimuli are considered safe, the cingulate cortex stimulates the perirhinal cortex and inhibits the BLA, thus mediating the cessation of defensive behaviors [152-154]. The increased activation of these cortical regions observed in CLF rats may be related to a disrupted attribution of emotional valence to external stimuli and, therefore, to an erroneous choice of defensive responses.

An early study with the Roman lines revealed no difference in Fos expression in the CeA, lateral parabrachial nucleus, or ventrolateral PAG in RHA or RLA rats. However, after CRH injections in the CeA, Fos expression was increased in the CeA of RLA animals and in the ventrolateral column of the PAG of both lines, which demonstrates the association between stress and behavioral responses of Romans [155]. Meyza *et al.* (2008) [156] also observed no differences in the expression of Fos in several brain regions of the Roman lines in basal conditions. However, following exposure to acute stress or after some behavioral tests, such as the OF, EPM, and hole board, RLA rats showed an increase in Fos expression in the PVN, amygdala BLA, CeA, medial and cortical areas, mPFC, and hippocampus CA1, CA 2 and CA3 areas. These findings highlight the increased susceptibility of the RLA rats to stress and point toward a differential pattern of Fos expression to the one observed in the CHF line.

Differences in Fos expression induced by stressful events like behavioral tests have also been evaluated in the HAB/ LAB lines. Following innate fear tests, open arm challenge, and OF, HAB rats showed altered Fos responses in prefrontal-cortical, limbic, and hypothalamic areas, such as the paraventricular nucleus and the lateral and anterior regions. Additionally, differences were found in the medial preoptic area and subregions of the amygdala, hypothalamus, nucleus accumbens, midbrain, and pons. On the other hand, decreased expression in Fos was verified in the hypothalamus of LAB rats [157-159]. Following the forced swim test, HAB rats displayed an increase in Fos expression in the limbic areas such as the prelimbic cortex, parts of the amygdala, the BNST, dorsal hippocampus, dorsal lateral septum, areas of the hypothalamus, PAG, and locus coeruleus. These differences are attenuated with the chronic treatment of paroxetine. In LAB rats, for most of the same areas, the swim stress induces a smaller Fos response [160]. Moreover, Muigg *et al.* (2008) [161] mapped the Fos expression during the extinction of cued conditioned fear responses. Associated with deficits in the extinction of conditioned behavior, HAB rats showed an attenuated Fos response in the infralimbic and cingulate cortices as well as in the lateral amygdala, whereas increased Fos response in the medial part of the CeA. Noteworthy is that none of the aforementioned studies found any differences in the basal levels of Fos expression between HAB and LAB rats. Only when elicited by potentially aversive and stressful situations did differences become apparent, suggesting a close association between stress, altered brain activity, and innate patterns of the expression of defensive responses.

## 6 ENDOCRINE AND METABOLIC ASSAYS

As mentioned before, CHF rats are more susceptible to the effects of chronic stress, while CLF’s behavior remains unaltered [37]. This effect can be at least partially derived from alterations in the HPA axis, which is known to play a crucial role in stress control and is closely associated with anxiety disorders [142, 143]. Studies using the Carioca lines showed that CHF male rats also contain higher levels of plasma corticosterone when compared to both CLF and control animals [120, 162]. The stress caused by contextual fear conditioning provoked an increase in corticosterone in both CHF and control groups, but not in CLF animals [163]. Moreover, the expression of the glucocorticoid receptor (GR) was found to be significantly reduced in the DG of the CHF group [120]. The hippocampus plays an important inhibitory role in the regulation of the HPA axis [164, 165] and thus indirectly affects the modulation of glucocorticoids levels, like corticosterone. For instance, Brinks *et al.* (2009) [166] demonstrated that the increase in glucocorticoids is crucial in the formation of aversive memories. They showed that BALB/c mice, which display an increased acquisition of aversive memories in comparison to C57BL/6J ones, had a significantly higher release of corticosterone after training and extinction. In this sense, the results obtained from studies using the Carioca lines suggest that differences in stress responsivity may be related to phenotypic and genetic baseline conditions.

Studies with the Roman and the HAB/LAB lines found no difference in the basal concentration of corticosterone. However, after stress, RLA rats respond with a steeper increase of glucocorticoid in comparison to RHA animals [106, 167, 168]. Similarly, several studies showed that the increase in plasma corticosterone levels triggered by exposure to stressful events is greater in HAB than in LAB rats and that such an increase in plasma corticosterone levels is generally followed by an equivalent increase in adrenocorticotropic hormone (ACTH) [169-171]. ACTH is produced by the pituitary gland and stimulates the production and release of cortisol from the adrenal gland [172]. However, there has also been evidence that after social defeat, an increase in corticosterone levels is higher in LAB rather in than HAB rats [173]. Overall, these results support the concept that susceptibly to stress is likely associated with altered HPA axis stress responses.

Mousovich-Neto *et al.* (2015) [162] described other endocrine and metabolic differences between control and CHF male animals. Results showed that CHF rats exhibited larger retroperitoneal and epididymal fat depots, and higher levels of both serum triglycerides and total cholesterol, which in turn can be related to alterations in glucocorticoids processing and a decrease in testosterone and thyroid hormone levels. Glucocorticoids are important for preadipocyte differentiation, adipogenesis, and lipid storage [174, 175]. Also, mutations in GR genes can alter their affinity with glucocorticoids and promote increased serum triglycerides and total cholesterol levels [176]. Thus, the alterations observed in the corticosterone levels and the expression of GR in the hippocampus of CHF rats may be linked to differences in their body composition. Moreover, testosterone can inhibit pre-adipocyte maturation and decrease triglyceride accumulation *via* the activation of lipoprotein lipase. In addition, the thyroid hormone triiodothyronine (T3) can affect the catabolic lipoprotein pathway through modulation of the expression of low-density lipoprotein receptors in the liver and other tissues [177-180]. Therefore, the relation between high levels of corticosterone and the decrease in serum testosterone and T3 levels found in CHF animals can be connected to an increase in adiposity, serum triglycerides, and total cholesterol levels.

The low levels of T3 were also found to be linked to a decrease in brown adipose tissue type 2 (BAT) activity [162]. BAT, an important tissue for energy expenditure, depends on the local production of T3 to function. The hypofunction of this system can contribute to lower oxygen consumption in CHF rats. Finally, the study showed an increase in serum leptin levels and fasting glycemia in CHF animals. Unbalance in emotional expression has been associated with insulin resistance as glucocorticoid treatment impairs glucose uptake by visceral adipose tissue [181]. The authors, thus, suggest that the altered fear behavior of CHF rats may be associated with the development of a metabolic syndrome.

Besides data related to the HPA axis, to our knowledge little is known about endocrine differences in other breeding lines of anxiety-like behavior. Evidence obtained from studies using the Roman lines shows that although they possess similar basal levels of prolactin (PRL) after stress, RLA rats showed increased PRL reactivity, which is related to the management of emotional responses and the “controllability” of the stressor [182]. This line also displayed an increase in total cholesterol, but triglyceride levels were similar to levels observed in RHA rats [183]. PRL was also found to be augmented in HAB rats in combination with an increase in oxytocin levels [171, 184], a neuropeptide known to be critically involved in the regulation of emotional behavior [185-187]. Moreover, even though overall levels of vasopressin (AVP) were similar in HAB and LAB rats, AVP expression and release in the PVN of HAB rats were found to be higher under both basal and stimulated conditions [188]. As already mentioned, the PVN plays an important role in fear processing.

Differences in oxidative stress between the Carioca lines have also been evaluated. Hassan *et al.* (2013) [34] showed that the levels of oxygen reactive species and lipid peroxidation are increased in the cortex, hippocampus, and cerebellum of CHF in comparison to CLF male rats. This characteristic can be associated with a decrease in the activity of antioxidant enzymes - catalase (CAT), and glutathione peroxidase (GPx) - in the CHF line. Moreover, the injection of diphenyl diselenide, a compound with antioxidant, glutathione peroxidase-mimetic, and neuroprotective properties induced an increase in freezing responses in CHF female animals, while reducing freezing in CLF rats [189]. Previous studies showed the association between oxidative stress and anxiety-like behavior in rodents. Oxidative stress induced by either a high-palatable diet or xanthine promoted anxiety-like behavior in rodents [190, 191]. On the other hand, treadmill exercise and antioxidant treatment were shown to reduce oxidative stress in rat brain regions implicated in anxiety responses and have an overall anxiolytic effect [192]. In the same line, oxidative damage to specific brain regions has been linked to anxiety-like behavior. Vitamin A administration induced lipid peroxidation and protein carbonylation in the hippocampus and fear responses in the dark-light exploration task of rodents [193]. Yet, a high-salt diet during the postweaning period of male Wistar rats induced less activity of antioxidant enzymes and higher levels of lipid peroxidation in the amygdala as well as a decreased expression of anxiety-like responses in the EPM and OF tests [194]. Reduction of neuroinflammation and oxidative stress in the PFC of mice was observed following treatment with curcumin, which also induced an improvement in the spatial working memory and anxiety-like behavior of the animals [195]. Moreover, in a heterogeneous sample of Swiss albino male mice, higher levels of anxiety correlated were found to be with increased oxidative stress [196].

## CONCLUSION

Since the first publication in 2008, growing and compelling evidence from behavioral studies has placed the CHF rats as an animal model of generalized anxiety disorder. Tables **1** and **2** summarize the methods and main results obtained over the years using the Carioca lines. In comparison to CLF rats, CHF animals show increased anxiety-like behavior in response to contextual and cued fear conditioning as well as in innate fear paradigms such as the EPM and the OF. CHF animals also show higher stress-related responses in the FST and after exposure to chronic stress compared to CLF rats. We describe and discuss important alterations in components of the fear circuitry identified in CHF rats that could account for these behavioral responses. Neurochemical and functional differences in the amygdala, hippocampus, cingulate cortex, limbic cortices, and the role of serotoninergic mediation give support to the enhanced fear-related responses seen in the CHF line. Moreover, metabolic and endocrine findings point out a poor and dysfunctional regulation of the physiological stress system in these animals. Finally, we also highlight recent data on the association between alterations of the dopaminergic circuitry and the impulsive and hyperactive behavior of the CLF rats. Overall, this review presents a broad picture of the current findings in the Carioca lines and supports their importance for the study of psychiatric disorders.

## Figures and Tables

**Fig. (1):**
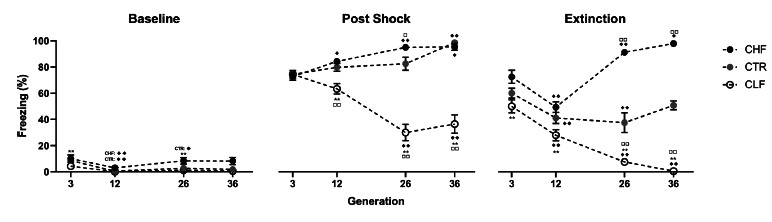
Freezing in context-conditioned fear experiments in the 3^rd^, 12^th^, 26^th^, and 36^th^ generations of the Carioca lines. Lines and circles are means ± SEM. Control rats (CTR; black circle), Carioca high-conditioned freezing (CHF; gray circle), Carioca low-conditioned freezing (CLF; empty circle). When no error bar is visible, they are smaller than the circle. *P < 0.05, and **P < 0.01 are displayed for comparison between CHF and CLF lines within the same generation. ^□^*P* < 0.05, and ^□ □^ *P* < 0.01 are displayed for comparison between either of the Carioca lines and CTR rats within the same generation. ^◆ ◆^ *P* < 0.01, ^◆ ◆^ *P* < 0.01 are displayed for comparison between third generation and sequent ones. The generations displayed here have been previously analyzed by Gomes & Landeira-Fernandez., 2008 [21], Gomes *et al*., 2011 [22], Cavaliere *et al.*, 2020 [25], and Lages *et al.*, 2021 [26].

**Table 1:** Description of the methods used in the studies with Carioca lines.

**Author (Year)**	**Rat Line**		**Generation**	**Sex**	**Age/Body Weight**	**Drugs/Intervention**	**Behavioral Tests**	**Biochemical/Neurochemical Analyses**
Gomes & Landeira-Fernandez (2008) [21]	▪ CHF ▪ CLF		3^rd^	Male	-	Bilateral electrolytic lesions of the amygdala	Contextual fear conditioning	Histology analysis in order to localize the electrolytic lesions
Dias *et al.* (2009) [33]	▪ CHF ▪ Control	▪ 4^th^ ▪ 6^th^		Male	▪ 2-3 months old ▪ 254-379 g (CHF) ▪ 250-394 g (Control)	-	▪ Elevated plus maze ▪ Social interaction test ▪ Forced swim test ▪ Object recognition test	Qualitative and quantitative analyses of the dorsal hippocampal tissue (dentate gyrus, CA1 and CA3 areas)
Galvão *et al.* (2011) [96]	▪ CHF ▪ CLF		9^th^	Male	6 months old	Electrical stimulation of the dPAG	Contextual fear conditioning	Histology analysis in order to localize the positions of the electrode tips
Gomes *et al.* (2011) [22]	▪ CHF ▪ CLF ▪ Control		12^th^	▪ Male ▪ Female	75-80 days old	-	Contextual fear conditioning	-
Hassan *et al.* (2013) [34]	▪ CHF ▪ CLF		▪ 7^th^ ▪ 12^th^ ▪ 16^th^	Male	▪ 15-20 weeks old ▪ 350-480 g	-	▪ Contextual fear conditioning ▪ Elevated plus maze	Analyses of the cortex, hippocampus, and cerebellum by: ▪ Total ROS production (DCF-RS) ▪ Rate of lipid peroxidation (TBARS) ▪ GPx and CAT activity
León *et al.* (2013) [163]	▪ CHF ▪ CLF ▪ Control		11^th^	-	-	-	Contextual fear conditioning	Plasma corticosterone
Salviano *et al.* (2014) [35]	▪ CHF ▪ CLF		10^th^	▪ Male ▪ Female	▪ 2-3 months old ▪ 190-330 g	-	▪ Elevated plus maze ▪ Forced swim test ▪ Contextual fear conditioning	-
Gomes *et al.* (2014) [197]	▪ CHF ▪ CLF		8^th^	Male	▪ 12-20 months old ▪ 350-450 g	-	Contextual fear conditioning	-
Dias *et al.* (2014) [120]	▪ CHF ▪ Control	▪ 9^th^ ▪ 13^th^ ▪ 14^th^		Male	2-3 months old	Three BrdU i.p. injections every 16 h (100 mg/kg at 10 mg/mL) in a separate group	▪ Morris water maze ▪ Passive avoidance test	▪ Immunostaining and quantification of the DG: immunohistochemistry (BrdU, Ki-67 and DCX) and immunofluorescence (GR expression) ▪ Dendritic spine density ▪ Serum corticosterone ▪ BDNF expression ▪ GABA release
Réus *et al.* (2014) [115]	▪ CHF ▪ CLF ▪ Control		4^th^	Male	▪ 2-3 months old ▪ 254-379 g (CHF) ▪ 250-394 g (Control)	Chronic mild stress (CMS)	-	Analyses of the prefrontal cortex, hippocampus, amygdala and nucleus accumbens by: ▪ Comet assay ▪ Immunoblotting (levels of NMDAR1, NMDAR2A, NMDAR2B, EAAT1 and EAAT2)
Mousovich-Neto *et al.* (2015) [162]	▪ CHF ▪ Control	-		Male	▪ 2-3 months old ▪ 254-379 g (CHF) ▪ 250-394 g (Control)	-	Contextual fear conditioning	▪ Evaluation of food intake, body weight, and fat depots ▪ Fasting glycemia and glucose tolerance test ▪ Oxygen consumption ▪ Serum hormone quantification (corticosterone, testosterone, T3, T4, TSH, leptin, and insulin) ▪ Serum triglycerides and total cholesterol ▪ BAT D2 activity
Hassan *et al.* (2015) [189]	▪ CHF ▪ CLF		17^th^	Female	▪ 15-20 weeks old ▪ 180-300 g	▪ Diphenyl diselenide (10, 50, or 100 mg/kg/i.p.) ▪ Canola oil (vehicle)	Contextual fear conditioning	-
León *et al.* (2017) [32]	▪ CHF ▪ CLF		▪ 10^th^ ▪ 15^th^ ▪ 20^th^	Male	4-6 months old	▪ Ketanserin tartrate 97% (0.5 mg/kg/i.p.) ▪ Ketanserin tartrate 97% (5 nmol/µl/IL and PL cortices) ▪ 2% DMSO (vehicle)	▪ Contextual fear conditioning ▪ Elevated plus maze	-
Bezerra-Karounis *et al.* (2017) [198]	▪ CHF ▪ CLF ▪ Control		▪ 30^th^ ▪ 31^st^	▪ Male ▪ Female	-	Oral consumption of: ▪ Alcohol (2%, 4%, 6%, and 10%) ▪ Quinine (2 μM) ▪ Saccharin (7.5 mM)	Contextual fear conditioning	-
Macêdo-Souza *et al.* (2020) [24]	▪ CHF ▪ CLF ▪ Control		28^th^	Male	13-17 weeks old	-	Auditory fear conditioning	-
León *et al.* (2020) [137]	▪ CHF ▪ CLF ▪ Control		-	Male	▪ 3-4 months old ▪ 190-330 g	-	Contextual fear conditioning	Fos protein immunochemistry in different brain structures
Cavaliere *et al.* (2020) [25]	▪ CHF ▪ CLF ▪ Control		▪ 24^th^ ▪ 25^th^ ▪ 26^th^	Male	240-300 g	▪ Midazolam (.25, .5, .75, and 1.0 mg/kg/i.p. - volume 1.0 ml/kg) ▪ Saline (vehicle)	▪ Contextual fear conditioning ▪ Elevated plus maze	-
Lages *et al.* (2021) [23]	▪ CHF ▪ CLF ▪ Control		36^th^	Male	75 days old	-	Contextual fear conditioning	-
Lages *et al.* (2021) [37]	▪ CHF ▪ CLF		-	Male	110 days old	Chronic unpredictable mild stress (CUMS)	▪ Contextual fear conditioning ▪ Open field ▪ Forced swim test ▪ Elevated T-maze	-
Goulart *et al.* (2021) [38]	▪ CHF ▪ CLF ▪ Control		▪ 18^th^-20^th^ ▪ 21^st^-23^rd^ ▪ 26^th^ ▪ 27^th^	Male	▪ 3-5 months old ▪ 250-335 g	-	▪ Contextual fear conditioning ▪ Hot plate test ▪ Open field ▪ Y-maze ▪ Forced swim test	Western blot and immunofluorescence of: ▪ AMPAR (GluA1 and GluA2) ▪ NMDAR (GluN1, GluN2A and GluN2B) ▪ PSD-95
Lages *et al.* (2021) [84]	▪ CLF ▪ Control	-		Male	90 days old	▪ Haloperidol (0.5, 1.0, and 1.5 mg/kg/i.p.) ▪ Methylphenidate (0.5, 1.0, and 2.0 mg/kg/i.p.) ▪ Saline (vehicle)	▪ Contextual fear conditioning ▪ Evaluation of catatonia ▪ Open field	-
Lages *et al.* (2022) [26]	▪ CHF ▪ CLF		32^nd^	Male	110 days old	▪ Chronic unpredictable mild stress (CUMS) ▪ High-sugar/high-fat (HSHF) diet supplementation	▪ Contextual fear conditioning ▪ Open field ▪ Forced swim test	-

**Abbreviations**: BrdU: bromodeoxyuridine; CMS: chronic mild stress; CUMS: chronic unpredictable mild stress; dPAG: dorsal periaqueductal gray; HSHF: high-sugar/high-fat diet.

**Table 2:** Description of the main results obtained in the studies with Carioca lines.

**Author (Year)**	**Results**
Gomes & Landeira-Fernandez (2008) [21]	▪ Histology showed induced damage, usually symmetrical; ▪ Bilateral electrolytic lesions affected most of the BLA, CeA, lateral portions of the amygdala and small portions of the ventral striatum; ▪ ↑ freezing in CHF than CLF; ▪ Amygdaloid lesions ↓ the percentage of conditioned freezing in the CHF and CLF.
Dias *et al.* (2009) [33]	▪ ↓ Number of entries and percentage of time spent in the open arms in CHF at the EPM; ▪ ↑ Anxiety scores and ↓ time exhibiting active social interactive behavior in CHF at the social interaction test; ▪ CHF did not differ from control rats in this depressive behavior paradigm at the FST; ▪ No significant differences were observed in the object recognition test, qualitative and quantitative histological analysis.
Galvão *et al.* (2011) [96]	▪ Histology indicated that electrode tips were located inside the dPAG; ▪ ↑ Aversive freezing and escape thresholds in CHF than CLF; ▪ CHF animals expressed ↑ dPAG-evoked post-stimulation freezing behavior than CLF.
Gomes *et al.* (2011) [22]	▪ ↑ Freezing in male than female across all the lines; ▪ ↑ Amount of conditioned freezing in CHF, ▪ Control presented intermediate levels of freezing; ▪ ↓ Freezing behavior in CLF.
Hassan *et al.* (2013) [34]	▪ ↑ Freezing in CHF than CLF across all three generations; ▪ CHF showed a ↓ percentage of entries and time spent in the open arms in the EPM than CLF; ▪ ↑ DCF-RS emission intensity and TBARS levels in the hippocampus, cortex, and cerebellum in the CHF compared to CLF; ▪ ↓ CAT activity in the cortex and hippocampus in CHF than CLF; ▪ ↓ GPx in all three brains structures in CHF than CLF.
León *et al.* (2013) [163]	▪ ↑ Levels of freezing in CHF than CLF; ▪ Control exhibited an intermediate level of freezing; ▪ ↑ Corticosterone concentrations before and after the contextual fear conditioning in CHF than CLF and control.
Salviano *et al.* (2014) [35]	▪ ↓ Time spent in the open arms of the EPM in CHF than CLF; ▪ Females spent ↑ time in the open arms of the EPM when compared to males; ▪ No differences among groups were found in the FST; ▪ ↑ Freezing in males more than females in both the CHF and CLF lines; ▪ ↑ Freezing behavior in CHF than CLF.
Gomes *et al.* (2014) [197]	▪ ↑ Defensive freezing behavior in CHF than CLF during the phenotyping testing session; ▪ Differences between CHF and CLF were observed only in the first session block during, extinction and re-extinction; ▪ No significant differences were observed in the other session blocks.
Dias *et al.* (2014) [120]	▪ ↓ Distance traveled along trials in both CHF and control in MWM; ▪ ↓ Latency to enter the target zone and time spent in the target quadrant in CHF in the MWM; ▪ ↑ Latency to step down from the platform of the PAT in the CHF; ▪ ↑ Basal corticosterone levels in CHF; ▪ ↓ Expression of GR in the DG in CHF; ▪ No differences were found in the total estimated number of BrdU+ or Ki-67+ cells and no differences in cell survival between groups; ▪ DCX quantification revealed a ↓ in the number of neuroblasts in the DG in CHF; ▪ ↓ Dendritic arborization in CHF and no differences were seen in the length of primary dendrites or length of secondary dendrites; ▪ CHF displayed ↑ dendritic spine linear density compared to control; ▪ ↑ Expression of proBDNF and ↓ GABAergic inhibition in the CHF hippocampus.
Réus *et al.* (2014) [115]	▪ ↑ DNA damage index and frequency induced by stress and anxiety in the hippocampus, amygdala and nucleus accumbens; ▪ Any DNA damage index or frequency in the prefrontal cortex for all groups tested; ▪ NMDAR1: ↑ in the prefrontal cortex, hippocampus and amygdala in the CHF, but a ↓ in the CHF+CMS; ▪ NMDAR2: ↓ in the amygdala in CHF and CMS groups, ↑ in CHF+CMS, ↓ in CLF in the nucleus accumbens, and no differences in the prefrontal cortex or hippocampus; ▪ NMDAR2B: ↑ in the hippocampus in CHF and CLF, ↓ in the amygdala in CHF+CMS and CLF+CMS, and ↑ in the prefrontal cortex and nucleus accumbens in CHF; ▪ EAAT1: ↑ in the prefrontal cortex in CLF+CMS, ↓ in the hippocampus in all groups, ↑ in the amygdala in CHF and CLF+CMS, and no alterations were found in the nucleus accumbens; ▪ EAAT2: ↑ in the prefrontal cortex in CHF, ↑ in the hippocampus in CHF and ↓ in CLF+CMS, ↓ in the amygdala in CHF, and no alterations were observed in the nucleus accumbens.
Mousovich-Neto *et al.* (2015) [162]	▪ No differences in body weight and food intake were found in both groups; ▪ ↑ Retroperitoneal and epididymal fat depots in CHF; ▪ ↑ Fasting glycemia in CHF and glucose tolerance test was unaffected in both groups; ▪ ↓ Energy consumption over 24 h in CHF and locomotor activity was similar between both groups; ▪ ↑ Serum levels of corticosterone, leptin, total cholesterol, and triglycerides in CHF; ▪ ↓ Serum levels of testosterone and T3 in CHF; ▪ No differences were found in serum levels of T4, TSH, and insulin in both groups; ▪ ↓ BAT D2 activity in CHF compared with control.
Hassan *et al.* (2015) [189]	▪ ↑ Freezing in CHF than CLF; ▪ ↑ Conditioned freezing in CHF that received 10 mg/kg diphenyl diselenide compared with CLF that received the same dose; ▪ ↓ Reliable conditioned freezing in CHF that received 50 mg/kg diphenyl diselenide compared with CHF vehicle; ▪ ↑ Conditioned freezing in CLF that received either 50 or 100 mg/kg diphenyl diselenide compared with CLF vehicle.
León *et al.* (2017) [32]	▪ Systemic ketanserin ↑ percentage and time spent on the open arms in the EPM in CHF compared with the vehicle; ▪ Ketanserin IL microinjections ↑ percentage and time spent on the open arms in the EPM in CHF, and ↓ percentage of time spent freezing in CHF but ↑ freezing in CLF animals; ▪ Ketanserin PL microinjections ↑ percentage of open arms entries in CHF and had no effect in CLF, and ↓ conditioned freezing in CHF compared with vehicle and had no effect in CLF.
Bezerra-Karounis *et al.* (2017) [198]	▪ ↑ Freezing observed in males than females across all three levels of the breeding line factor; ▪ ↑ Conditioned freezing in CHF line and ↓ in the CLF; ▪ ↑ Bodyweight in males than females and between CHF groups compared to CLF; ▪ ↓ Bodyweight in males from all lines and females CHF during the forced alcohol 10% consumption; ▪ ↓ Saccharin intake in male and female CHF; ▪ ↑ Quinine consumption in female CHF and CLF compared to male; ▪ ↑ Forced alcohol intake in females among all lines and ↑ in male CHF; ▪ No differences in alcohol intake among groups at the 2% and 4% alcohol concentrations; ▪ ↓ Consumption of alcohol at 6% in male CLF and ↑ in female CHF; ▪ ↑ Alcohol intake at 10% in both male and female CHF compared to the other lines.
Macêdo-Souza *et al.* (2020) [24]	▪ Day 1: No differences observed at baseline freezing between all experimental groups, and ↑ freezing in CS acquisition in all groups that received footshocks paired with the auditory stimuli (Tone + Shock); ▪ Day 2: ↑ Freezing in CHF Tone + Shock groups than CTL and CLF Tone + Shock; ▪ Day 3: Similar baseline values in CHF Tone + Shock and CHF Tone only. All Tone + Shock groups exhibited ↑ overall freezing compared to both their respective baseline values and their Tone-only counterparts, and CHF exhibited ↑ freezing than CLF.
León *et al.* (2020) [137]	▪ ↑ Conditioned freezing in CHF line and ↓ in the CLF; Fos expression: ▪ ↑ PL in control compared to both CHL and CLF; ▪ ↑ CG1 and PR in CLF compared to CHF and control; ▪ ↑ LS and LC in CHF compared to CLF and control; ▪ ↑ MS and DG in control compared to both CHF and CLF; ▪ ↑ PVN and BLA in CHF and ↓ in CLF compared to control; ▪ No other significant differences were found.
Cavaliere *et al.* (2020) [25]	▪ ↑ Freezing response in CHF than both CLF and control in the pre-shock period, post-shock period, and test session; ▪ ↓ Percentage of entries and time spent in the open arms on the EPM in CHF and ↑ in CLF compared with control; ▪ ↓ Absolute number of closed arms entries in CHF compared to CLF and control; ▪ Midazolam ↑ percentage of open arms entries and time spent on the open arms in all groups, but in CLF it was only observed at the lowest dose tested (.25 mg/kg).
Lages *et al.* (2021) [23]	▪ ↑ Freezing in CHF in baseline measurements, and ↑ freezing response in CHF and control induced by footshock; ▪ In the first testing session, CHF exhibited maximum and CLF exhibited minimal freezing behavior; ▪ In the second testing session, after extinction protocol, CHF and control exhibited a ↓ in freezing, whereas CLF exhibited unaltered freezing; ▪ In all sessions, CLF did not exhibit significant freezing behavior.
Lages *et al.* (2021) [37]	▪ ↑ Freezing behavior before and after CUMS in CHF than CLF and control; ▪ ↓ Number of entries and time spent in the central area of the OF in CHF after CUMS; ▪ ↑ Mobility in CLF compared with CHF in both control and CUMS in the OF test; ▪ ↓ Latency to immobility in the re-exposure in control groups and ↑ in CUMS groups in the FST test; ▪ Baseline latencies were similar among all groups in the elevated T-maze; ▪ ↑ Avoidance latency in CHF groups and ↓ in CLF groups in the elevated T-maze; ▪ ↑ Escape latency from the open arms of the elevated T-maze in CHF+CUMS compared to CHF control and no alterations in CLF; ▪ ↑ Freezing during escape trials in CHF+CUMS compared to CHF control and CLF groups in the elevated T-maze.
Goulart *et al.* (2021) [38]	▪ ↑ Freezing behavior in CHF and ↓ in CLF compared to control; ▪ No significant differences in the response latency were observed at 47 ºC and 52 ºC in the hot plate test; ▪ ↑ Locomotor activity, total distance traveled, rearing behavior and time spent in the central area in CLF in the OF test; ▪ No significant differences in the percentage of entries into the new arm were observed between groups in the Y-maze test; ▪ ↓ Time spent in the new arm in CHF compared to CLF and control in the Y-maze test; ▪ ↑ Immobility time in CHF than CLF and control in the FST; ▪ No differences in both glutamatergic receptor subunits and PSD-95 levels were observed in the dorsal hippocampus among groups; ▪ ↑ GluN1 subunit in the ventral hippocampus in CHF and CLF rats compared with control; ▪ ↓ GluN2A levels in CHF and ↓ GluN2B levels in CLF in the ventral hippocampus compared to other groups; ▪ ↑ Ratio GluN2A/GluN2B in CLF and ↓ in CHF; ▪ ↑ GluA1 content in CHF and ↑ GluA2 content in CLF in the ventral hippocampus compared to other groups; ▪ ↓ Ratio GluA1/GluA2 in CLF compared to CHF and control.
Lages *et al.* (2021) [84]	▪ Experiment 1: Within each dose, haloperidol induced catatonia regardless of rat line. However, ↑ catatonia in control at 1.0 mg/kg dose and ↓ in CLF at the end-point (2 h) with the 0.5 mg/kg dose; ▪ Experiment 2: Methylphenidate induced ↑ freezing in CLF at 0.5 and 1.0 mg/kg doses compared to saline. ↑ number of crossings between peripheral squares in CLF at 2.0 mg/kg dose of methylphenidate; ▪ Experiment 3: No effect of methylphenidate doses on freezing was found but CLF showed ↓ freezing in the post-shock and test session.
Lages *et al.* (2022) [26]	▪ CHF were more susceptible to the effects of CUMS than CLF; ▪ ↓ Freezing behavior in CHF+HSHF group after the CUMS protocol; ▪ ↑ Freezing behavior in CLF, showing similar effects in both stress and diet; ▪ HSHF supplement had a ↑ protective effect over the behavioral alterations caused by stress in CHF compared to CLF; ▪ ↑ Number of entries in the center and the number of crossings in the periphery on the OF test caused by HSHF diet in both lines; ▪ ↑ Locomotory behavior in CHF+HSHF on the open field test; ▪ ↑ Latency to immobility and ↓ in total immobility times on the FST in both lines caused by CUMS and the opposite effect was observed in the HSHF diet groups.

**Abbreviations**: AMPAR: α-amino-3-hydroxy-5-methyl-4-isoxazolepropionic acid receptor; BAT D2: brown adipose tissue type 2 iodothyronine deiodinase; BDNF: brain-derived neurotrophic factor; BLA: basolateral amygdala; BrdU: bromodeoxyuridine; CAT: catalase; CeA: central nucleus of the amygdala; CG1: anterior cingulate cortex-subregion 1; CHF: Carioca High-conditioned Freezing; CLF: Carioca Low-conditioned Freezing; CMS: chronic mild stress; CUMS: chronic unpredictable mild stress; DCF-RS: dichlorofluorescein reactive species; DCX: doublecortin; DG: dentate gyrus; dPAG: dorsal periaqueductal gray; EPM: elevated plus maze; FST: forced swim test; GABA: gamma-aminobutyric acid; GPx: glutathione peroxidase; GR: glucocorticoid receptors; HSHF: high-sugar/high-fat diet IL: infralimbic cortex; LC: locus coeruleus; LS: lateral septum; MS: medial septum; MWM: morris water maze; NMDAR: N-methyl-D-aspartate receptor; OF: open field; PAT: passive avoidance test; (PhSe)_2_: diphenyl diselenide; PL: prelimbic cortex; PR: perirhinal cortex; PSD-95: postsynaptic density-95; PVN: paraventricular nucleus of the hypothalamus; ROS: reactive oxygen species; TBARS: thiobarbituric acid reactive substances; TSH: thyroid stimulating hormone.

## LIST OF ABBREVIATIONS

ACTH: Adrenocorticotropic Hormone
ADHD: Attention-deficit/Hyperactivity Disorders
ASST: Attentional Set-shifting Test
BLA: Basolateral Amygdala
BNST: Bed Nucleus of the Stria Terminalis
CeA: Central Amygdala
CRH: Corticotropin-releasing Hormone
CS: Conditioned Stimulus
CUMS: Chronic Unpredictable Mild Stress
DG: Dentate Gyrus
DRN: Dorsal Raphe Nucleus
EPM: Elevated Plus Maze
FST: Forced Swim Tests
GAD: Generalized Anxiety Disorder
GR: Glucocorticoid Receptor
IL: Infralimbic
LC: Locus Coeruleus
OF: Open Field
PAG: Periaqueductal Gray
PL: Prelimbic Cortices
SHR: Spontaneously Hypertensive Rats
TH: Tyrosine Hydroxylase
VEGF: Vascular Endothelial Growth Factor
